# The Relationship between Type 2 Diabetes and Platelet Indicators

**Published:** 2017-09

**Authors:** Xiangyu CHEN, Le FANG, Hongbo LIN, Peng SHEN, Tao ZHANG, Hui LI, Xiaoyong LI, Min YU, Chunxiao XU, Jie ZHANG, Feng LU, Xiaofu DU, Ruying HU, Jieming ZHONG

**Affiliations:** 1. Zhejiang Provincial Center for Disease Control and Prevention, Hangzhou, P.R. China; 2. Yinzhou District Center for Disease Control and Prevention, Ningbo, P.R. China; 3. Ningbo Municipal Center for Disease Control and Prevention, Ningbo, P.R. China

**Keywords:** Diabetes mellitus, Mean platelet volume, Platelet distribution width, Platelet count

## Abstract

**Background:**

Currently, the studies on the relationship between type 2 diabetes and platelets indicators were carried out on a selective small sample population generally. Large sample studies in this area are rare, especially in Chinese population. In this study, we aimed to investigate the relationship between type 2 diabetes and the mean platelet volume (MPV), platelet count (PLT) and platelet distribution width (PDW).

**Methods:**

This is a cross-sectional analysis of the routine health examination data from 20128 participants with complete baseline data in Yinzhou District 2013. The detection of indicators in this study was completed by automatic hematology analyzer.

**Results:**

The PDW and PLT were not significantly different between diabetic group and non-diabetic group, (16.00% vs. 16.00%, *P*=0.88, and 194*10^9^/L vs. 196*10^9^/L, *P*=0.05 respectively). The MPV was significantly higher in diabetic group (9.3fl vs. 9.2fl, *P*<0.05). MPV was an independent risk factor of diabetes mellitus (Unadjusted OR=1.07 (95% CI: 1.03, 1.11), Adjusted for age, sex OR=1.07 (95% CI: 1.02, 1.12)). The adjusted odds ratio of diabetes rose with increasing MPV levels and were most pronounced in subjects with MPV levels exceeding the 90th percentile (MPV ≥10.70 fl, Crude or=1.23 adjusted or=1.19).

**Conclusion:**

There was no relationship between the presence of diabetes with PDW and PLT. The MPV was independently associated with the presence of diabetes.

## Introduction

The platelets play significant roles in the integrity of normal homeostasis and atherosclerosis process (1, 2). They are also closely associated with cardiovascular events. There were numerous studies on the role of platelets in cardiovascular events during last decades, many of them focused on thrombotic complications. The thromboxane generation increased in patients with thrombotic complications (3), especially in the cardiovascular diseases patients with poor glycemic control (4). Diabetes mellitus (DM) has been considered as a ‘prothrombotic state’ with enhanced platelet reactivity (5), researchers found the morphological changes of platelets and the increased platelet activity occurred in diabetic patients (6).

Among the Chinese adults, the estimated prevalence of diabetes was 11.6% while the prevalence of prediabetes was 50.1% (7). Taking the total number of Chinese populations into consideration, there were approximate 113.9 million diabetes patients and 493.4 million people with prediabetes (7), indicating that diabetes has become a major public health problem in China. Higher MPV values have recently been shown in patients with proliferative diabetic retinopathy (8). Since microvascular, complications of DM are important causes of morbidity and medical expenditure, to determine indicators of these complications such as MPV will be highly beneficial.

Currently, the studies on the relationship between platelet and diabetes were carried out on a selective small sample population generally. Large sample studies in this area are rare, especially in Chinese population.

This study was conducted to explore the relationship between selected platelet indicators (MPV, PDW, PLT) and prevalence of diabetes.

## Materials and Methods

This study was carried out in Yinzhou District; Ningbo 2013. Totally, 20128 subjects come from a random sample of 7 towns in the district. The data of the subjects come from the local health information platform. In this study, we defined diabetes patients as fasting glucose ≥126mg/dl, 2h postprandial glucose ≥200mg/dl, or treatment with insulin or oral hypoglycemic drugs. The dataset of 20128 subjects having all non-missing values for diabetes, platelet indicators, was defined as the complete case dataset and was used for domain analysis. The level of significance (α) was kept at 5%. Odds ratios and 95% confidence interval were estimated by logistic regression, the selection of confounding factors in logistic regression model refer to the similar study in this field (1). Because the goodness-of-fit tests for normal distribution of these indicators show that the indicators were not normally distributed (*P*<0.01), the median (interquartile (IQR)) for the indicators was used in the descriptive analyses (1). In regression analyses on the indicators, however, the continuous indicators were used according to the central limit theorem because the sample size is quite large (9).

## Results

### Characteristics of subjects and platelet indicators information according to diabetic status

The median age of the subjects was 62 yr old (IQR 55–69 yr old). Other characteristics of subjects were presented in Table 1.

**Table 1:** Demographic and baseline information of the participants according to diabetic status

**Character**	**DM**	**Non-DM**	***P***
Age (Median + IQR)	66.00 (61.00–71.00)	62.00 (53.00–68.00)	<0.01
Age group (yr) (%)			
18–44	1.25	11.38	<0.01
45–59	16.62	27.64	<0.01
≥60	82.13	60.98	<0.01
Sex ratio	875:1598	8009:9646	<0.01
(Male: Female)			
Education level (%)			
Illiteracy	12.80	9.44	<0.01
Primary School	67.44	58.56	<0.01
Middle School	17.91	27.18	<0.01
High School	1.68	4.00	<0.01
College and above	0.17	0.82	<0.01
Marital status (%)			
Unmarried	0.56	2.79	<0.01
Married	90.67	90.78	0.87
Divorced	0.13	0.60	<0.01
Widowed	8.64	5.83	<0.01
Occupation (%)			
Farmer	8.63	45.16	<0.01
Worker	53.63	32.65	<0.01
Housework/Retired	37.74	22.19	<0.01
MPV (Median + IQR)	9.30 (8.70–10.00)	9.20 (8.60–9.90)	<0.01
PLT (Median + IQR)	194.00 (161.00–231.00)	196.00 (164.00–231.00)	0.05
PDW (Median + IQR)	16.00 (15.70–16.40)	16.00 (15.60–16.40)	0.88
HDL cholesterol (Median + IQR)	1.25 (1.07–1.53)	1.26 (1.07–1.51)	0.83
Triglycerides (Median + IQR)	1.43 (1.04–2.07)	1.46 (1.05–2.09)	0.20
RBC (Median + IQR)	4.41 (4.12–4.69)	4.45 (4.17–4.77)	<0.01
WBC (Median + IQR)	5.90 (5.00–7.00)	5.60 (4.70–6.60)	<0.01
Hemoglobin (Median + IQR)	132.00 (124.00–141.00)	134.00 (125.00–145.00)	<0.01
RDW (Median + IQR)	12.60 (12.00–13.40)	12.60 (12.00–13.40)	0.05
Hematocrit (%) (Median + IQR)	39.40 (37.40–42.00)	40.10 (37.90–43.00)	<0.01
MCV (Median + IQR)	91.90 (88.40–95.20)	92.40 (89.00–96.00)	<0.01
MCH (Median + IQR)	30.30 (29.20–31.30)	30.50 (29.50–31.50)	<0.01
MCHC (Median + IQR)	333.00 (325.00–342.00)	334.00 (326.00–342.00)	<0.01

Demographic features listed including age, sex, education level, marital status and occupation

Data are presented as median (IQR) or percent. *P* values were obtained by linear regression and chisq test

Of the 20128 subjects, 2473 had diabetes; the proportion was 12.29%. The proportion of hypertension was 47.76%. There were 7780 subjects with hypertriglyceridemia; the proportion was 38.65%. There were 4200 subjects with low HDL cholesterol, the proportion was 20.87%.

Table 2 reports the baseline and platelet indicators information in subjects with and without diabetes. MPV was significantly higher in the subjects with diabetes (9.30 vs. 9.20 femtoliter (fL), *P*<0.01). Of all the subjects, those diagnosed with diabetes had elder age (P<0.01). The female proportion was higher in the diabetic group (*P*<0.01). There was no significant difference in PLT, PDW, HDL cholesterol and triglycerides between subjects with and without diabetes. The WBC was higher in the diabetic cases while the other indictors of CBC were higher in the nondiabetic cases (Table 1).

**Table 2:** Linear association of fasting serum glucose with platelet indicators

**Variable**	**Estimated parameters**	**SE.**	***t***	***P***
**Fasting serum glucose**				
**Age**	0.13	0.001	18.38	<0.01
**Triglycerides**	0.11	0.008	15.27	<0.01
**PDW**	0.05	0.005	7.49	<0.01
**PLT**	0.06	0.000	6.94	<0.01
**MPV**	0.03	0.009	3.54	<0.01
**HDL cholesterol**	0.001	0.008	0.08	0.94

Estimated parameters stands for standardized beta

SE. stands for standard error // *P*-values were obtained by linear regression

### Linear association of fasting serum glucose with platelet indicators

Table 2 reports the linear association of fasting serum glucose with platelet indicators and the confounding factors. The fasting serum glucose was positively correlated with age, triglycerides, PDW, PLT and MPV. There was no linear association of fasting serum glucose with HDL cholesterol (Table 2).

### MPV in different characteristic group of subjects

Table 3 reports the MPV in different characteristic group of subjects. The MPV was significantly different with and without diabetes. MPV was significantly higher in the female subjects (9.30 vs. 9.20 fL, *P*<0.01). MPV was significantly higher in the subjects with Low HDL Cholesterol (9.30 vs. 9.20 fL, *P*<0.01). MPV was significantly higher in the subjects without hypertriglyceridemia (9.30 vs. 9.20 fL, *P*<0.01) (Table 3).

**Table 3:** MPV in different characteristic group of subjects

**Character**	**YES (Median + IQR)**	**NO (Median + IQR)**	***P***
**Diabetes**	9.30 (8.70–10.00)	9.20 (8.60–9.90)	<0.01
**Hypertension**	8.90 (8.40–9.56)	9.60 (8.90–10.40)	<0.01
**Male**	9.20 (8.50–9.90)	9.30 (8.60–10.00)	<0.01
**Low HDL Cholesterol**	9.30 (8.60–10.10)	9.20 (8.60–9.90)	<0.01
**Hypertriglyceridemia**	9.20 (8.60–9.80)	9.30 (8.60–10.00)	<0.01

Data are presented as median (IQR). *P-*values were obtained by linear regression

### The diabetes prevalence by each MPV quartile

To understand better, the relationship between MPV and diabetes prevalence, we divided the MPV into quartiles. As shown in Table 4, the diabetes prevalence increased with increasing MPV quartiles (The difference of diabetes prevalence was compared by chi-square trend test) (Table 4).

**Table 4:** The diabetes prevalence by each MPV quartile

**Quartile**	**DM**	**Non-DM**	**Prevalence (%)**	***P***
**1^st^**	607	4927	10.97	<0.01
**2^nd^**	583	4220	12.14	
**3^rd^**	607	4184	12.67	
**4^th^**	676	4324	13.52	

*P*-value was obtained by chi-square trend test

### Odds ratio of diabetes with elevated MPV levels

The multivariable model was applied to quantitatively assess the risk the MPV brings to the diabetic prevalence. The unadjusted odds ratio was 1.07, the 95% confidence interval was (1.03, 1.11). After adjusting age, sex, hypertension, PDW, PLT, HDL cholesterol and Triglycerides, the odds ratio was 1.07 and the 95% confidence interval was (1.02, 1.12).

As shown in Table 5, when compared with the subjects with the MPV level less than 50th (<9.20 fl), the odds ratio of having diabetes rose with increasing MPV levels. In a multivariable model adjusted for age, sex, the adjusted odds ratios were obtained. The adjusted odds ratios were lower than the unadjusted ones (Table 5).

**Table 5:** Odds ratio of diabetes with elevated MPV levels^*^

**Cut point percentile**	**MPV fl**	**Diabetes %**	**Unadjusted OR (95%CI)**	***P***	**Adjusted OR1 (95%CI)**	***P***
**<50th**	<9.20	11.41	1.00		1.00	
**≥50th**	≥9.20	13.07	1.17 (1.07–1.27)	<0.01	1.17 (1.06–1.28)	<0.01
**≥75th**	≥9.90	13.39	1.20 (1.09–1.33)	<0.01	1.18 (1.06–1.33)	<0.01
**≥90th**	≥10.70	13.70	1.23 (1.07–1.42)	<0.01	1.19 (1.02–1.40)	0.03

* The *P*-values of OR in the table were obtained by comparison with MPV level less than 50th

## Discussion

DM is a complex disease characterized by chronic hyperglycemia responsible for complications affecting the kidneys, eyes, peripheral nerves, and micro and macrovascular systems. The reported prevalence of diabetes in these 7 towns was 3.89% while the diabetes proportion among the subjects was 12.29%. The main cause of this difference might be the subjects are older and the diabetes patients focus more on their health.

This is the largest study so far to explore the relationship between the platelet indicators and diabetes in a large unselected population at present in China.

In this study, the platelet indicators, known as PDW and PLT, were not significantly different between diabetic patients and non-diabetic population.

The MPV is a marker of platelet size and an easily measured platelet indicator, which increase during platelet activation. In this study, the MPV was significantly higher in the diabetic patients. The prevalence of diabetes increased with the increasing MPV levels. This is consistent with other studies that have also reported the increase in MPV in diabetic patients in comparison with non-diabetic controls (1,2,10,11). The mechanism why the MPV were higher in diabetes patients was not yet fully understood (1). The hyperglycemia status in diabetes patients cause the osmotic swelling and lead to the increased MPV finally (12). The insulin may cause the generate of larger platelets by megakaryocytes (13). The clear mechanism of this relationship needs to be explored in the further study.

### Limitations of our study

In this study, we only find the relationship between type 2 diabetes and platelet indicators, the clear mechanism of this relationship still needs to be explored. Also, this relationship in type 1 diabetes has not involved.

MPV was associated with diabetes prevalence. The adjusted odds ratio was lower than the unadjusted one because of the uneven distribution of the confounding factors, known as age and sex.

## Conclusion

There was no relationship between type 2 diabetes with PDW and PLT. The MPV was independently associated with type 2 diabetes.

## Ethical considerations

Ethical issues (Including plagiarism, Informed Consent, misconduct, data fabrication, double publication and/or submission, redundancy, etc.) have been completely checked by the authors.
